# Cellular immunotherapy for hematological malignancy: recent progress and future perspectives

**DOI:** 10.20892/j.issn.2095-3941.2020.0801

**Published:** 2021-08-05

**Authors:** Zhengli Xu, Xiaojun Huang

**Affiliations:** 1 Peking University People’s Hospital, Peking University Institute of Hematology, National Clinical Research Center for Hematologic Disease, Beijing Key Laboratory of Hematopoietic Stem Cell Transplantation, Beijing 100044, China

**Keywords:** Cellular immunotherapy, hematologic, CAR-T, NK, stem cell transplantation

## Abstract

Advancements in the field of cellular immunotherapy have accelerated in recent years and have changed the treatment landscape for a variety of hematologic malignancies. Cellular immunotherapy strategies exploit the patient’s immune system to kill cancer cells. The successful use of CD19 chimeric antigen receptor (CAR) T-cells in treating B-cell malignancies is the paradigm of this revolution, and numerous ongoing studies are investigating and extending this approach to other malignancies. However, resistance to CAR-T-cell therapy and non-durable efficacy have prevented CAR-T-cells from becoming the ultimate therapy. Because natural killer (NK) cells play an essential role in antitumor immunity, adoptively transferred allogeneic NK and CAR-modified NK cell therapy has been attempted in certain disease subgroups. Allogenic hematopoietic stem cell transplantation (allo-HSCT) is the oldest form of cellular immunotherapy and the only curative option for hematologic malignancies. Historically, the breadth of application of allo-HSCT has been limited by a lack of identical sibling donors (ISDs). However, great strides have recently been made in the success of haploidentical allografts worldwide, which enable everyone to have a donor. Haploidentical donors can achieve comparable outcomes to those of ISDs and even better outcomes in certain circumstances because of a stronger graft *vs.* tumor effect. Currently, novel strategies such as CAR-T or NK-based immunotherapy can be applied as a complement to allo-HSCT for curative effects, particularly in refractory cases. Here, we introduce the developments in cellular immunotherapy in hematology.

## Introduction

In recent decades, the treatment model for hematologic diseases has undergone extensive changes. Traditionally, the management of hematological malignancies has relied on chemotherapy regimens, local radiotherapy, and palliative support care, which lead to poor prognosis and high mortality^1^. However, with advances in the knowledge of tumor pathophysiology, therapies are continually evolving. In particular, the emergence and development of cellular immunotherapies have revolutionized the field of hematologic tumor treatment. Cellular immunotherapies harness and augment the natural capability of the immune system to fight malignant diseases, through protocols involving harvesting immune cells, expanding them, or redirecting them to target cancer cells^2^. Currently, cellular immunotherapies play an essential role in the treatment of patients with various hematological malignancies.

In the field of hematological malignancy treatment, the representative cellular immunotherapies mainly include chimeric antigen receptor T cell (CAR-T) therapy, natural killer (NK) cell-based immunotherapy, and allogeneic hematopoietic stem cell transplantation (allo-HSCT). CAR-T cell therapy, one of the most highly publicized and promising advances in the past few years, involves engineering T cells to express chimeric antigen receptors (CARs)^3^. NK cell-based immunotherapy is increasingly becoming recognized to have an important role in innate antitumor immunity^4^. Allo-HSCT, one of the oldest forms of cellular immunotherapy, has provided a model of immunotherapy and remains the only curative option for hematologic malignancies^1^. Recently, great strides have been made on the basis of the success of haploidentical allografts worldwide, thereby allowing everyone to have a donor^5^. In this review, we discuss recent cutting-edge treatments and prospects for future directions of the various cellular therapy products under development.

## CAR-T cell therapy: an incredibly promising area for treating hematologic malignancies

Adoptive T-cell therapy with CAR-expressing T-cells has emerged as one of the most promising cellular immunotherapy modalities, showing remarkable antitumor efficacy in the treatment of hematologic tumors. This therapy involves targeting tumor antigens directly as well as augmenting different targeted immune effectors^6^. Given the initial efficacy of CAR-T technology in B-cell malignancy, expanding applications to the treatment of other hematological diseases, such as multiple myeloma, T-cell leukemia/lymphoma, and myeloid malignancy, has been of great interest (**Table 1**)^7^.

**Table 1:** Selected results of clinical studies on CAR-T-cells

Study	Disease	Target	CAR dose (× 10^6^/kg)	Patient No.	Efficacy	Survival/duration	CRS
Maude et al. 2014	R/R B-ALL	CD19	0.76–20.6	30	CR 90%	6 m EFS 67%, 6 m OS 78%	27% (severe)
Lee et al. 2014	ALL/NHL	CD19	0.03–3.6	21	CR/CRi 66.7%	OS 51.6% at 9.7 months	76% (total)
Neelapu et al. 2017	NHL	CD19	2	101	CR 54%	OS 52% at 18 months	13% (≥ grade 3)
Fry et al. 2018	R/R B-ALL	CD22	0.3–3	21	CR 57%	Median duration: 6 months	76% (total)
Pan et al. 2019	R/R B-ALL	CD22	0.02–3.47	34	CR/CRi 70.5%	1-year LFS 71.6%	91% (total)
Raje et al. 2019	MM	BCMA	50–800 × 10^6^ (total)	33	CR 45%	Median PFS: 11.8 months	76% (total)
Cohen et al. 2019	MM	BCMA	10–500 × 10^6^ (total)	25	OR 20%–64%	Median PFS: 2.2–4.2 months	88% (total)
Wang et al. 2017	HL	CD30	11–21	18	OR 39%	Median PFS: 6 months	11% (≥ grade 3)
Ramos et al. 2017	HL/ALCL	CD30	20–200 × 10^6^/m^2^	9	CR 33%	CR duration: 9–36 months	NA
Wang et al. 2020EHA	T-ALL	CD7	6–15	5	CR 80%	NA	100%

CR, complete response; CRS, cytokine release syndrome; EFS, event-free survival; OS, overall survival; OR, objective response; PFS, progression free survival.

### Promising results of CAR-T cells for B-cell malignancy

#### CD19 CAR-T cells: the paradigm of the CAR-T revolution

To date, CD19 has been the most extensively applied and most successful target of CAR-T therapy. CD19 CAR-T cells have produced promising outcomes in the treatment of B-cell acute lymphoblastic leukemia (B-ALL), with complete remission (CR) rates as high as 90% and deep molecular responses in patients with relapsing/refractory (r/r) disease^8,9^. These encouraging results prompted the approval of CAR-T19 for children and young adults with r/r B-ALL by the U.S. Food and Drug Administration in 2017^10^.

CARTs targeting CD19 have also induced sustained antitumor immune responses in patients with B-cell non-Hodgkin lymphoma (NHL) and chronic lymphocytic leukemia (CLL)^11^, although the response rates vary for different disease subtypes^12–14^. The reported CR rates were 43%–54% in large B-cell lymphoma and 21%–29% in CLL^14^. In particular, in a cohort of 101 patients with r/r NHL, the objective response and CR rates have been reported to be 82% and 54%, respectively. The overall survival was 52% at 18 months. On the basis of these promising outcomes, CAR-T19 has also been approved for the treatment of adults with r/r large B cell lymphoma^13^.

Because CD19-directed CAR-T-cells are a remarkable innovation for treating hematologic malignancies, numerous reports have reviewed and summarized their current status, challenges, and potential future applications^11,15^; therefore, we will not provide more details in this regard.

#### Other potential target CARs in B-cell malignancy

To overcome CD19-negative relapses in B-cell malignancy, other B-cell specific antigens (CD22 and CD79b) are being investigated as potential targets. CD22 is expressed in most cases of B-ALL and is usually retained after CD19 antigen loss. In a recent study using second-generation CD22 CAR-T cells, whereas most patients with B-ALL previously failed CD19 CAR-T therapy, the use of CD22 CAR-T cells resulted in CR rates of 73% (11/15) among patients who received ≥ 1 × 106 CD22-CAR-T cells/kg^16^. In another CD22 CAR-T cell study in China, 34 B-ALL patients relapsed after CD19 CAR-T cell therapy, whereas 70% CR rates (24/34) were achieved after CD22 CAR-T cell therapy^17^. CD79b is an important mediator of the development and maintenance of mature B cells, and CD79b CAR-T cells have also been investigated. Recent findings have demonstrated that CAR-T cells targeting CD79b alone or in combination have promise in treating B cell lymphomas, as demonstrated *in vitro* and in xenograft models of lymphoma^18^.

### Extending CAR-T cells to other hematologic malignancies

The successful use of CAR-T cells in B-cell malignancies has encouraged the extension of this approach to other malignancies, such as multiple myeloma (MM), Hodgkin lymphoma (HL), T-cell malignancy, and acute myeloid leukemia (AML) (**Table 2**). Clinical efficacy varies according to the disease subtype and specific antigens.

**Table 2:** The potential target CARs of T cells

Disease type	Potential targets
B cell malignancy	CD19, CD20, CD22, CD79b, CXCR5…
T-cell malignancy	CD7, CD5, CD4, CD30, CDTRBC1…
Multiple myeloma	BCMA, CD38, CD138, SLAMF7, CD44v6…
AML	CD33, CD123, CD117, CLL-1…
Hodgkin lymphoma	CD30…

#### BCMA directed CAR-T cell therapy in multiple myeloma

B-cell maturation antigen (BCMA) is specifically expressed in MM cells in most patients with MM and has recently been indicated to be a promising antigen for CAR-T cells against MM^19^. Selected BCMA CAR-T-cell clinical trials have demonstrated overall response rates of 43%–100% at various doses with various CAR construct specifications^20^. Currently, bispecific CAR-T-cells targeting BCMA and CD38^21^ or BCMA and CD19^22^ are also under investigation and have shown encouraging outcomes.

#### CD30 directed CAR-Ts in HL

In HL, clinical trials of CD30-directed CAR-Ts have been ongoing for several years. In a phase I clinical trial, CAR-T-30 cell infusion yielded a 39% (7/18) objective response for patients with relapsed or refractory HL^23^. Ramos et al.^24^ have reported the outcomes of 9 patients with relapsed/refractory CD30-positive lymphoma. Of 8 treated patients who had active disease at the time of infusion, 2 patients (25%) achieved CR. One treated patient who was already in CR at the time of infusion maintained a CR for more than 2 years. The efficacy of CD30-directed CAR-T cells is expected to be further enhanced by optimizing the lymphodepletion regimen, enhancing migration to the tumor sites, and combining this therapy with other immune regulators^25^.

#### CAR-Ts in T cell malignancy

T cell malignancies comprise a heterogeneous group of diseases, each reflecting a clonal evolution of dysfunctional T cells at various stages of development. To date, no specific antigen has been identified with universal expression on cancerous T cells, thus hampering CAR-T cell therapy for T cell malignancies^26^. Preclinical studies have shown that CD7 CAR-T cells have robust activity against T cell malignancies *in vitro* and *in vivo*^27^. A study from China has demonstrated the efficacy of CD7 CAR-T cells in treating r/r T-ALL, reporting that 80% (4/5) of patients achieved minimal residual disease (MRD)-negative CR at 1 month post-infusion. Clinical CD7 CAR trials in T cell malignancies are ongoing, and other promising targets, including CD5, CD4, CD30 and CDTRBC1, are being investigated. The clinical efficacy results are currently awaited.

#### CAR-T therapy in AML

The key problem in CAR-T cell treatment of AML is the absence of truly AML-specific surface antigens, because myeloid antigens are often coexpressed on normal hematopoietic stem/progenitor cells. Numerous CAR-T cell clinical trials are enrolling patients with AML, most of which involve targeting of CLL-1 (also known as CLEC12A), CD33, or CD123; however, the clinical efficacy of these therapies remains uncertain^28^. Further development and optimization of CAR-T therapy for patients with AML are warranted.

### Will CAR-T cells be the ultimate therapy for hematologic malignancies?

CAR-T cell therapy has provided promising advances in hematologic malignancy. However, to achieve the goal of making this the ultimate therapy, several hurdles must be overcome, including 1) resistance to CAR-T cell therapy; 2) non-durable efficacy; 3) an apparent lack of suitable targets as effective as CD19 in non-B-cell malignancies; and 4) unavoidable and unpredictable toxicity.

#### Mechanisms of resistance to CAR-T cell therapy and potential solutions

We consider resistance to CD19 CAR-T cell therapy as an example, because CD19 CAR-T cells have long been applied in many cases. Two patterns of post-CAR relapse exist: CD19-positive relapse and CD19-negative relapse^29^. For CD19-positive relapse, CD19 remains present on the surface of cancer cells and can be detected by flow cytometry. The key mechanism of relapse is the poor persistence of CAR-T-cells^29^. For CD19-negative relapse, CD19 is absent, and cancer cells therefore evade CAR-mediated recognition and clearance despite CAR-T-cell persistence^29^.

Improving CAR-T-cell persistence is a major strategy to address CD19-positive relapse. Laboratory strategies consist of optimizing CAR structure (including the extracellular domain, transmembrane domain, and intracellular domain of CAR), genome editing of CAR-T-cells to “delete” inhibitory receptors on the surfaces of CAR-T-cells, and designing artificial antigen-presenting cells to periodically activate CAR-T-cells^29,30^. In clinical practice, human-derived CAR-T-cells apply a novel humanized single-chain variable fragment and have been demonstrated to have great persistence and killing ability *in vitro* and *in vivo*. In a pilot phase I study, 18 patients with r/r ALL with or without prior murine CD19 CAR-T therapy were administered humanized CD19-targeted CAR-T cells^31^. Among the 14 patients without previous CAR-T therapy, 13 (92.9%) achieved CR or CRi, and 1 of the 3 patients who failed a second murine CAR-T infusion achieved CR after humanized CAR-T infusion. The LFS rate was 71.4% at day 180. Another strategy in clinical settings is adding chemotherapeutic agents before CAR-T-cell infusion. A meta-analysis has found that patients who received a lymphodepletion regimen before cell infusion achieved a 6-month PFS rate of 94.6%, whereas patients who did not receive a lymphodepletion regimen achieved a PFS rate of only 54.5% (*P* < 0.001)^32^. One possible mechanism is that the lymphodepletion regimen decreased the tumor burden, eradicated regulatory T-cells, and improved the activation of antigen-presenting cells^33^.

As a solution for CD19-negative relapse, dual/multi-targeted CAR-T-cells have been explored in both laboratory and clinical studies. Many research groups have attempted to develop dual-target CARs by targeting CD19 and another antigen simultaneously, such as CD22 or CD20. A phase 1 clinical trial has enrolled pediatric patients with r/r B-ALL, and administered bicistronic CAR-T cell therapy targeting CD19 and CD22. All 7 evaluable patients achieved CR/CRi (100%) as well as molecular negative remission after dual-target CAR infusion^34^. Another clinical trial in China using CD19/CD22 dual CAR-T cells has reported that all 7 patients with a median cell dose achieved CR, 6 of whom had MRD-negative CR^35^.

#### Unsatisfactory duration of efficacy in clinical use

Many patients ultimately relapse after CAR-T-cell therapy^7^. Despite the strikingly high CR rate, a high relapse rate after CAR-T19 therapy has emerged as one of the major problems as follow-up is prolonged. Researchers from the Memorial Sloan Kettering Cancer Center have reported a median event-free survival (EFS) of 6.1 months at a median follow-up of 29 months among patients with relapsed B-ALL who received CD19 CAR-T infusion. Moreover, 17 of 26 (65.4%) patients in CR who underwent no further therapy relapsed or died^36^. A more recent study from China has also shown unsatisfactory long-term efficacy in patients who received CD19 CAR-T cell therapy for r/r B-ALL after allo-HSCT. Chen et al.^37^ have reported a total CR rate of 85.7% in 34 eligible patients. However, with a median follow-up of 20.7 months, 17 patients had relapsed at a median of 4.5 months post-infusion. The cumulative recurrence rate at 18 months was as high as 68.3%^37^. Therefore, the efficacy of CAR-T cells is not durable, thus preventing it from becoming the ultimate therapy. Consequently, additional treatment, including bridged HSCT, must be optimized to further improve long-term efficacy post-CAR-T infusion.

#### Indication limitations and CAR-T cell therapy-related toxicity

As previously described, none of the targets are as attractive as CD19, because they are expressed on other critical hematopoietic cells and/or lack uniform expression on tumor cells. Therefore, CAR-T efficacy is limited for non-B-cell hematologic malignancies. Novel strategies aimed at increasing the therapeutic indication of CAR-T cells for hematologic malignancies have been undertaken, such as tuning CAR affinity and antigen expression on malignant cells to increase specificity, targeting intracellular tumor-associated antigens, and targeting the tumor microenvironment^3^. Preclinical findings have suggested that these options may be valid to increase the range of potential applications of CAR-T immunotherapy.

The main toxicities are cytokine-release syndrome (CRS), neurotoxicity (CAR-T-cell-related encephalopathy syndrome), and on-target off-tumor toxicity (B cell aplasia with CD19 CAR-T). CRS is the most frequent adverse event after CAR-T cell therapy, ranging in severity from low-grade constitutional symptoms to a high-grade syndrome associated with fatal multiple organ dysfunction^38^. The incidence of CRS is as high as 90%, and CRS is associated with disease burden, cell dose, and disease subtype^39^. Neurotoxicity is another common and unique toxicity after CAR-T cell therapy, which occurs in up to 67% of patients with leukemia and 62% of patients with lymphoma^40^. In the long term, almost all patients treated with CAR-T 19 experience B cell aplasia, an expected on-target off-tumor toxicity^41^.

#### CAR-T may serve as a complement to HSCT

CAR-T therapy is known to lead to a rapid and high response, and allo-HSCT remains the only curative option for hematologic malignancies. However allo-HSCT is poorly curative for patients with refractory or active disease at the time of transplantation. Thus, CAR-T therapy may serve as an effective way to induce remission and serve as a “bridge to transplant,” which could potentially further improve the outcomes of transplantation for patients with refractory and relapsed cancer^39^.

Data from an NIH study have reported that 10 of 12 B-ALL patients who became MRD-negative after CAR-T therapy went on to receive allogeneic transplantation, and no subsequent relapses were observed^9^. Another study from China has examined 42 refractory/hematological relapsed and 9 refractory MRD-positive B-ALL cases in patients who received CD19-targeted CAR-T cells. A total of 90% of patients with evaluable r/r B-ALL achieved CR/CRi, and 100% of the initially MRD-positive patients were found to have become MRD-negative. Twenty-three of 27 CR/CRi patients bridged to allo-HSCT remained MRD-negative during a median observation time of 133 days after allo-HSCT, whereas 9 of 18 CR/CRi patients without allo-HCT relapsed. These data demonstrate that CD19 CAR-T-cell therapy is an effective bridge to HSCT in patients with r/r B-ALL^42^.

In general, CAR-T-cell therapy is one of the most important breakthroughs for the treatment of hematologic malignancies, particularly B cell neoplasms. Despite the current successes, many challenges remain in this field. At present, CAR-T therapy can be applied as a complement to allo-HSCT but is not the ultimate choice.

## NK cell-based immunotherapy

NK cells are highly cytotoxic innate immune effectors that rapidly exert cytotoxicity against tumor cells without prior sensitization, thus making them appealing candidates for cellular immunotherapy. Notably, NK cells recognize their target cells in a classical major histocompatibility (MHC)-independent manner, and NK cell killing is not dependent on the expression of a single antigen. Therefore, NK cells kill their targets in a non-specific manner and consequently could provide an “off-the-shelf” product readily available for immediate clinical use^4^. With the progressive understanding of NK cell immunobiology and the advances in manipulative techniques, the field of NK cell-based immunotherapy in hematological malignancies has been expanding over the past several decades (**Table 3**). Herein, we introduce several patterns of NK cell-based immunotherapy, mainly focusing on adoptively transferred allogeneic NK cell and CAR-modified NK cell therapy.

**Table 3:** Selected results of clinical studies on NK cell-based immunotherapy

	Study	NK cell infusion regimen	Results
In non-transplant setting			
In advanced AML	Miller, 2014	Haplo-NK, activated with IL-2, Flu/Cy	Five of 19 (26%) patients achieved CR
	Bachanova, 2014	Haplo-NK, depletion of Tregs, activation of IL-2, Flu/Cy	Eight of 15 patients (53%) achieved remission at day 28, CR (*n* = 3), (CRp; *n* = 2), and CRi (*n* = 3)
	Cooley, 2019	Haplo-NK cells given with rhIL-15, Flu/Cy	Fourteen of 40 patients (35%) achieved CR/CRi
In MRD positive AML	Zhao, 2020	IL-21/4-1BBL-expanded NK cellchemotherapy with Flu/Cy or anthracyclines/Cy	Effective rates were 50% or 60% in Flu/Cy (*n* = 10) or anthracyclines/Cy (*n* = 10); DFS was clearly better in the NK group than in the historical group
As consolidation or maintenance	Jiang, 2019	IL-21/4-1BBL-expanded NK cell infusion during 4 to 7 courses of chemotherapy	The 3-year LFS was better in the NK group than in the control group (65.1% *vs.* 43.5%, *P* = 0.047)
	Nguyen, 2019	Haplo-NK, activated with IL-2, Flu/Cy	NK cells did not improve EFS (60.7% *vs.* 69.1%; *P* = 0.553) over chemotherapy alone
In transplant setting			
Post-HSCT	Choi, 2014	Donor-derived, IL-15 plus IL-21-stimulated CD3-depleted NK cells on days 14 and 21 post HSCT	Post-transplantation NK cells significantly decreased leukemia progression (74% to 46%, *P* = 0.038)
	Choi, 2016	Additional donor NK-cell infusions given on days 6 and 9 in addition to 14 and 21 post HSCT	Compared with the above study findings, an additional NK infusion on days 6 and 9 was not associated with less leukemia progression
Pre-HSCT	Lee, 2016	IL-2 activated NK cell infusion after conditioning chemotherapy and before stem cell infusion	Durable CR occurred in 5 of 21 patients
	Ciurea, 2017	mbIL21 *ex vivo* expanded donor-derived NK cells on days −2, +7, and +28	The incidence was significantly lower in the NK group than in the control group for CMV reactivation (30.8%) (70.4%, *P* = 0.01)

CRp, CR without platelet recovery; CRi, CR with incomplete recovery.

### Adoptively transferred allogeneic NK cells in a non-transplant setting

NK cells can be isolated, expanded, or produced *ex vivo* to be transferred in an autologous or allogeneic setting. Adoptive NK cell therapy refers to the introduction of *ex vivo* manipulated NK cells to patients for clinical use. In malignant diseases, autologous NK cell activity is inhibited largely because of the KIR ligand match, thus conferring limited clinical benefits^43^. However, NK cells from an allogeneic source have offered a promising choice for immunotherapy^44^. Use of adoptively transferred allogeneic NK cells has shown clinical benefits against several hematologic malignancies in numerous preclinical and clinical studies, particularly against AML^45^.

#### Allogeneic NK cells in advanced AML

Initially, interleukin-2 (IL-2) was used for *ex vivo* culture and the expansion of primary NK cells. Miller et al.^46^ first demonstrated the feasibility and safety of adoptively transferred NK cells into patients. In this trial, NK cells were derived from haploidentical-related donors, activated with IL-2, and administered to lymphodepleted patients with AML. Finally, 5 of 19 (26%) patients with poor-prognosis AML conditioned with high-dose cyclophosphamide (Cy) and fludarabine (Flu) achieved CR. Over the years, modifications to this approach have led to remarkable progress. Bachanova et al.^47^ have reported that depletion of host regulatory T cells (Tregs) with IL-2-diptheria toxin enhances NK cell expansion and improves the efficacy of haploidentical NK cell therapy for refractory AML, with increased CR rates exceeding 50%. Thus, IL-2–activated allogeneic NK cell therapy is effective in advanced AML, but the use of IL-2 is limited by concurrent stimulation of immunosuppressive host Tregs.

IL-15 has also been identified as a candidate for potentiating the activation of NK cell therapy. In one study, 42 patients with r/r AML received either intravenous or subcutaneous recombinant human IL-15 (rhIL-15) after lymphodepleting chemotherapy and haploidentical NK cells. The overall rate of CR/CRi was 35% among 40 evaluable cases. A high frequency of CRS (56%) and neurotoxicity (33%) after treatment with subcutaneous rhIL-15 was observed, and the development of CRS had no effect on the disease response^48^.

Another way to produce large numbers of cytotoxic NK cells for immunotherapy is through a clonal NK cell line, such as NK-92. Boyiadzis et al.^49^ have reported that the adoptive transfer of NK-92 cells for the treatment of patients with r/r AML resulted in 3 of 6 patients showing transient clinical responses. Although limited data exist on the efficacy of this approach in AML, NK-92 cells have the advantages of easy expansion and can be repeatedly infused in the context of lymphodepletion.

#### Allogeneic NK cells in MRD-positive AML

More recently, Zhao et al.^50^ have explored the activity of clinical-grade membrane-bound IL-21/4-1BBL-expanded NK cell products against MRD-positive AML. In this study, clinical-grade NK cell expansion was induced by incubation of fresh peripheral blood mononuclear cells with equal numbers of irradiated membrane-bound IL-21/4-1BBL-expressing K562 cells for 2–3 weeks. In a mouse model, expanded NK cells were found to be effective in controlling K562 tumor growth *in vivo*. In the clinical cohort, the effective rates were 50% or 60% in MRD-positive patients who received chemotherapy with either Flu/Cy (*n* = 10) or anthracyclines/Cy (*n* = 10) before NK infusion, respectively. Compared with the case-paired matched control group, the NK cell infusion group had better disease-free survival (*P* = 0.017). The promising efficacy results suggest that NK cell therapy might be better at clearing a low tumor burden.

#### Allogeneic NK cells as consolidation or maintenance therapy in AML

Recently, Wang et al.^51^ have evaluated the efficacy of consolidation chemotherapy combined with NK cell infusion in low- or intermediate-risk AML. Overall, 45 NK cells were injected into 23 patients during 4 to 7 courses of chemotherapy. The 3-year LFS was better in the NK group than in the control group (65.1% *vs.* 43.5%, *P* = 0.047).

The clinical efficacy of adoptively transferred NK cells as a maintenance therapy in AML has led to conflicting outcomes. In an earlier pilot study, 10 children and young adults who had completed chemotherapy and were in the first CR of AML were enrolled. All participants received Cy/Flu, followed by KIR–human leukocyte antigen (HLA)-mismatched NK cells. With a median follow-up time of 964 days, all patients remained in remission, and the 2-year EFS estimate was 100%^52^. However, a phase II trial has reported that NK cell transfer as a maintenance therapy for pediatric AML in the first CR did not decrease relapse^53^. This trial included 21 children with intermediate-risk AML who were in the first CR after chemotherapy. A median of 12.5 × 106 purified, unmanipulated NK cells/kg were infused into children after Flu/Cy lymphodepletion. The lack of benefit might have been due to insufficient numbers and the limited persistence of alloreactive NK cells, and the observations do not preclude the treatment’s potential effectiveness. Currently, the outcomes of another phase II trial (registration No. NCT02763475) administering a higher number of NK cells are pending^54^.

### Allogeneic NK cells play a complementary role in the transplant setting

One of the most promising characteristics of alloreactive NK cells is their ability to lead to graft-*vs.*-leukemia effects without causing graft *vs.* host disease (GvHD). In addition, allogeneic NK cells can directly kill recipient T cells, thus leading to improved engraftment. In transplant settings, the infusion of allogeneic NK cells has been explored as a viable option to promote engraftment or prevent relapse. NK cells can be safely administered before and/or post-transplantation in patients with different types of hematological diseases.

#### Allogeneic NK cells administered post-HSCT

Passweg et al.^55^ first demonstrated that NK cells are well tolerated and beneficial in facilitating engraftment and inducing GVT effects without contributing to GvHD when used as adoptive immunotherapy in recipients of haploidentical HSCT. Later, Choi et al.^56^ investigated the clinical effects of IL-15 plus IL-21-stimulated CD3-depleted NK cells (median dose of 2.0 × 108/kg) given 2 and 3 weeks after HSCT in patients with hematological malignancies (*n* = 41). The outcomes from this cohort were compared with those of a historic cohort of 31 patients who underwent only HLA haploidentical HSCT. Less disease progression was observed in the HSCT plus NK group than in the HSCT only group (46% *vs.* 74%, *P* = 0.038). In a subsequent study, additional donor NK-cell infusions were given on days 6 and 9, in addition to 14 and 21 post-HSCT. Of 51 patients, 24/51 (47%) had 4 NK infusions. Of 45 evaluable patients, the 3-year OS rate was 9% in AML, 21% in ALL, and 9/45 in aGvHD. The updated results, compared with those from a previous study, showed that early administration of NK cells after HSCT caused substantial toxicity without improving antileukemic effects^56,57^.

#### Allogeneic NK cells administered before HSCT

In a phase I study, 21 patients with high-risk myeloid malignancy were treated with escalating doses of third-party NK cells from a haploidentical related donor after conditioning chemotherapy and before stem cell infusion from an HLA-matched donor. The infusion of haploidentical alloreactive NK cells was well tolerated and did not interfere with engraftment or increase the rate of GvHD after allo-HSCT. Durable CR occurred in 5 patients at high risk of disease relapse^58^.

Another phase I study has reported that multiple doses of NK cells (days −2, +7, and +28), which were expanded *ex vivo* with K562-mbIL21-41BBL feeder cells, can effectively prevent leukemia relapse^59^. Eleven of 13 enrolled patients with myeloid malignancy received all 3 planned NK cell doses (1 × 10^5^ to 1 × 10^8^/kg). No infusional reactions or dose-limiting toxicity occurred. All patients were engrafted with donor cells. One patient died of TRM, and one patient relapsed; all the others were alive and in remission at the last follow-up (median 14.7 months). The control group of patients consisted of all 45 people treated in the previous clinical trial with the same conditioning regimen but without NK cells. The incidence of CMV reactivation in the control group was significantly higher (70.4%) than that in the NK group (30.8%, *P* = 0.01).

Therefore, adoptively transferred NK cells can persist *in vivo* and mediate antitumor effects among AML patients in non-transplant or transplant settings. Currently, the timing of the NK cell infusion, NK cell dosage, and optimized pre-infusion conditioning regimens are critical factors that must be more extensively studied to assess the safety and efficacy of allogeneic NK cell infusions.

### Engineered NK: CAR-NK cell therapy

The introduction of CARs into NK cells aims to enhance their potent killing activity against cancer cells. These CARs recognize specific antigens on tumor cells and boost the natural killing of NK cells, on the basis of their expression of activating receptors^60^. As compared with CAR-T cells, CRA-NK cells have unique characteristics. NK cells have a shorter lifespan and secrete a safer cytokine profile than T cells; therefore, they might decrease the incidence and severity of adverse effects associated with autoimmunity and CRS. However, their limited persistence* in vivo* has also led to concerns regarding their potential efficacy^43^.

To date, primary human NK cells derived from peripheral blood, CB, or hematopoietic stem cell progenitors, as well as NK cell lines, have been successfully engineered to express CARs against several targets on hematological cancer cells. CAR-modified NK cell development has been largely limited to the preclinical stage, and studies have shown promising results for the use of CAR NK cells *in vitro* and in animal models against some hematological malignancies. Preclinical studies have shown that CD4/CD5 in T cell malignancy, CD19/CD20 in B cell malignancy, CD138 in MM, and CD3 in AML are promising targets showing good results^45,61^.

Data on the clinical use of CAR-NK have rarely been reported. Tang et al.^62^ have tested the safety and efficacy of CD33-CAR NK cells in 3 patients with r/r AML. This phase I study did not demonstrate clear clinical efficacy, yet this first-in-human clinical trial has shown that this therapy can be safely used in patients with r/r AML with a high tumor burden. At doses as high as 5 × 10^9^ cells per patient, no significant adverse effects were observed. In another phase 1 and 2 trial, HLA-mismatched anti-CD19 CAR NK cells derived from cord blood were administered to 11 patients with r/r CD19-positive B cell malignancy. The cells were expanded *ex vivo* and given in a single infusion at one of 3 doses (1 × 10^5^, 1 × 10^6^, or 1 × 10^7^ CAR NK cells/kg) after lymphodepleting chemotherapy. Among 11 patients with relapsed or refractory CD19-positive cancers, 8 (73%) had a response without the development of major toxic effects^63^.

In summary, NK cell-based immunotherapy holds great promise as a novel cellular immunotherapy against refractory hematologic malignancies. Genetically modified NK cells, such as CAR NK cells, have opened a new path to improving the efficacy of NK cell therapy. Notably, NK cells may provide an off-the-shelf therapy that can be applied as an allogeneic product to treat patients, thus eliminating the need for a personalized and patient-specific product.

## Allo-HSCT: the oldest form of cellular immunotherapy but the curative option

Allo-HSCT, the original immune-based cellular therapy, remains the only potentially curative treatment option for patients with hematologic malignancies. The general principles of allo-HSCT involve the following key points. First, allo-HSCT enables the rescue of patients following a conditioning regimen with administration of potentially myeloablative doses of chemotherapy and radiation. Second, it exerts a significant graft-*vs.*-tumor response that potentiates the eradication of malignant cells. Thus, allo-HSCT reconstitutes hematopoietic cell lineages with normal cells capable of continuous self-renewal and can confer long-term, disease-free survival to patients. With the broadening clinical indications and the application of alternative donors, its clinical use has been increasing each year^1^.

### History of allo-HSCT and its obstacles

The first allogeneic transplantation was performed in 1968 by E. Donnall Thomas, who later won the Nobel Prize for pioneering this technology. Subsequently, the development of allo-HSCT was hindered by major transplantation-related complications, including graft failure, GvHD, and prolonged immunodeficiency. With better understanding of HLA, improved transplantation techniques and the availability of novel drugs, allo-HSCT from identical siblings has yielded encouraging results and has been recommended as the standard choice for high-risk hematologic malignancy^64^. However, only 30% of patients in need of an allogeneic transplant have an HLA-genotypically identical sibling donor (ISD), thus providing an impetus for identifying other potential donors, including haploidentical donors (HIDs) and unrelated donors.

### Great progress in haplo-HSCT has opened a new era: everyone has a donor

Haploidentical family donors, such as parents, children, or haploidentical siblings, provide the benefits of rapid and nearly universal donor availability. Initially, an unacceptably high incidence of graft failure and severe GvHD occurred because of the HLA barrier, thereby hindering the development of haplo-HSCT for several decades^65^. However, in the past 2 decades, researchers worldwide have established several haplo-HSCT protocols based on different approaches to induce immune tolerance.

#### Representative approaches in haplo-HSCT

Three main methods are used for haplo-HSCT^66^. The first method is T cell depletion-based regimens, which originated from the Perugia group in Italy^67^. The second method is granulocyte colony stimulating factor (G-CSF) plus antithymocyte globulin (ATG)-based regimens with unmanipulated T cell replete grafts, which originated from the Peking group in China^68^. Finally, the third method is post-transplantation cyclophosphamide (PT-Cy)-based regimens with unmanipulated T cell replete grafts, which originated from the Baltimore group in the USA^69^. Because of the difficult manipulation techniques and high expense of T cell depletion *in vitro*, application of the first method has gradually decreased in recent years. Consequently, Professor Bacigalupo from Italy has commented that the “Beijing Protocol from China and PT-Cy Protocol from the US are currently the 2 major haploidentical platforms worldwide,” in 2020 EMBT E-Learning. The reliable protocols for haplo-HSCT have evolved from a “romance of the three kingdoms” to competition between China and the US.

#### Comparable outcomes of transplantation from HIDs and ISDs

The transplantation outcomes from haploidentical donors have been greatly improved with the advent of novel conditioning regimens, improved GvHD prophylaxis, and advances in supportive care (**Table 4**). Numerous studies have demonstrated that haplo-HSCT can achieve comparable results to those from matched related donor transplantation in different subtypes of hematologic malignancies, either in haplo-SCT based on immune tolerance induced by G-CSF and ATG modalities^70–72^, or in haploidentical allografts with PT-Cy settings^73^.

**Table 4:** Comparison of HIDs with ISDs in hematologic malignancies

Study	Disease	No. (HID *vs*. ISD)	Protocol in HIDs	Survival and relapse
Wang, 2015	AML in CR1	231 *vs*. 219	G-CSF and ATG	3-year DFS 74% *vs.* 78% (*P* = 0.34); relapse 15% *vs.* 15% (*P* = 0.98)
Wang, 2016	ALL in CR1	103 *vs.* 83	G-CSF and ATG	3-year DFS 61% *vs.* 60%, (*P* = 0.91), relapse 18% *vs.* 24% (*P* = 0.30)
Wang, 2016	MDS	226 *vs.* 228	G-CSF and ATG	4-year RFS 58% (HLA 3/6) *vs.* 63% (HLA 4–5/6) *vs.* 71% (ISD) (*P* = 0.14)
Ghosh, 2016	Lymphoma	180 *vs.* 807	PT-Cy based	3-year PFS 48% *vs.* 48% (*P* = 0.96), disease progression (37% *vs.* 40%, *P* = 0.51)
Chang, 2017	AML MRD+	56 *vs*. 20^#^	G-CSF and ATG	4-year LFS 80% *vs.* 48% (***P* = 0.007**), relapse 13% *vs.* 36% (***P* = 0.017**)
Chang, 2020	ALL MRD+	169 *vs.* 39	G-CSF and ATG	3-year LFS 65% *vs.* 43% (***P* = 0.023**), relapse 23% *vs.* 47% (***P* = 0.006**)
Yu, 2019	High-risk AML	83 *vs.* 106	G-CSF and ATG	3-year RFS 63% *vs.* 43% (***P* = 0.035**), relapse 14% *vs.* 24% (*P* = 0.101)

^#^Extracted from the prospective cohort. The bold values refer to “*P* <0.05” with significance.

Huang’s group has led several disease-specific, multi-center studies comparing outcomes between HIDs by using the Beijing Protocol and ISD HSCT in AML, ALL and myelodysplastic syndrome (MDS). In patients with intermediate- or high-risk AML in the first complete remission (CR1), a prospective, multi-center study was designed^70^ in which a total of 450 patients were assigned to receive HID (*n* = 231) or ISD HSCT (*n* = 219) according to donor availability. With a median follow-up of 952 days for surviving patients, the 3-year DFS (74% *vs.* 78%, *P* = 0.34), OS (79% *vs.* 82%, *P* = 0.36), incidence of relapse (15% *vs.* 15%, *P* = 0.98), and non-relapse-mortality (13% *vs.* 8%, *P* = 0.13) were all similar between HID and ISD recipients. In patients with Philadelphia-negative high-risk ALL in CR1, a biological phase 3 randomized, multi-center study has been conducted^71^ in which a total of 186 patients received transplants from ISDs (*n* = 83) or HIDs (*n* = 103). The 3-year DFS from CR (61% *vs.* 60%, *P* = 0.91), relapse rates (18% *vs.* 24%, *P* = 0.30), and non-relapse-mortality (13% *vs.* 11%, *P* = 0.84) did not differ between the HID and ISD groups. In patients with MDS, a multi-center, registry-based comparison^72^ has reported the outcomes of 454 MDS patients who underwent HSCT from HIDs (*n* = 226) or ISDs (*n* = 228). Among the 3/6 HID (*n* = 136), 4–5/6 HID (*n* = 90), and ISD patient groups, the 4-year adjusted OS values were 58%, 63%, and 73% (overall *P* = 0.07), respectively, and those of relapse-free survival were 58%, 63%, and 71% (overall *P* = 0.14).

In patients with lymphoma, the results of haplo-HSCT using PT-Cy with ISD-HSCT have also been compared^73^. The Center for International Blood and Marrow Transplant Research evaluated 987 adult patients undergoing either HID-HSCT (*n* = 180) or ISD-HSCT (*n* = 807) after reduced-intensity conditioning regimens. The median follow-up of survivors was 3 years. For HID-HSCT *vs*. ISD-HSCT, the 3-year rates of non-relapse mortality (15% *vs.* 13%, *P* = 0.41), relapse/disease progression (37%* vs.* 40%, *P* = 0.51), progression-free survival (48% *vs.* 48%, *P* = 0.96), and OS (61% *vs.* 62%, *P* = 0.82) were similar.

### Haplo-HSCT exerts a stronger GVT effect in both a clinical cohort and mouse model

With the improved management of transplant complications, disease relapse has become the main cause of transplant failure. In recent years, increasing evidence supports that haplo-HSCT may have a stronger graft-*vs*.-leukemia (GVL) effect than ISD-HSCT, because mismatches for HLA antigens on leukemic cells would provide alloimmune targets^74^. An earlier single-center study from our group has indicated that patients with r/r leukemia who received HID-HSCT experienced a significantly lower incidence of relapse than those who underwent ISD-HSCT (26% *vs.* 49%, *P* = 0.008)^75^. Recently, the stronger GVL effect from haplo-HSCT has been further demonstrated in more clinical cohorts and in animal models.

Positive pre-transplantation MRD is a risk factor for disease relapse but can be overcome by haploidentical allografts^76,77^. A retrospective study (*n* = 339) and a prospective study (*n* = 340) have been performed to compare the effects of pre-transplantation minimal residual disease (pre-MRD) on outcomes in patients with AML who underwent ISD-HSCT or who received unmanipulated haploidentical allografts^76^. In both the retrospective (*n* = 65) and the prospective study (*n* = 76), pre-MRD-positive participants receiving haplo-HSCT experienced a lower incidence of relapse than those who underwent ISD-HSCT (*P* < 0.001 and *P* = 0.017, respectively). Subsequently, Huang’s group designed a prospective genetically randomized study to evaluate donor options for pre-transplantation patients with MRD-positive ALL who underwent HID (*n* = 169) or ISD-HSCT (*n* = 39)^77^. Compared with the HID-HSCT cohort, the ISD-HSCT cohort had a higher 3-year incidence of relapse (47% *vs.* 23%, *P* = 0.006), and a lower probability of LFS (43% *vs.* 65%, *P* = 0.023) and OS (46% *vs.* 68%, *P* = 0.039). Another prospective multi-center cohort study has investigated the GVL efficacy of haploidentical donors compared with ISDs for high-risk AML in CR1. Overall, 189 patients were enrolled and assigned to groups transplantation from HIDs (*n* = 83) or ISDs (*n* = 106) according to donor availability (biological randomization)^78^. Although the TRM, DFS, and OS were similar between groups, the cumulative incidence of post-MRD positivity was 18% and 42% in the HID and ISD groups, respectively (*P* < 0.001).

More recently, Huang’s group has demonstrated a stronger GVL effect from haploidentical allografts than MHC matched allografts in animal models^79^. Two non-irradiated leukemia mouse models that carried the human AML-ETO or MLL-AF9 fusion gene were used to establish haploidentical and MHC-matched transplant models. Haplomatching the MHCs of leukemia cells with recipient mouse T cells prolonged leukemic mouse survival and decreased the leukemia burden. The stronger GVL effect in the haplo-HSCT group was mainly induced by decreased apoptosis and increased secretion of cytotoxic cytokines, including tumor necrosis factor-α, interferon-γ, pore-forming proteins, and CD107a secreted by T cells or NK cells. In addition, in a prospective clinical trial enrolling 135 AML patients with t(8;21) who showed positive MRD before transplantation, haplo-HSCT, compared with ISD-HSCT, has been observed to slow the kinetics of leukemia burden *in vivo* and decrease the incidence of relapse. *Ex vivo* experiments showed that cytotoxic T lymphocytes from the haplo-SCT group had higher cytotoxicity than those from the ISD-HSCT group 1 year after transplantation.

### Cellular immunotherapy in treating transplantation complications

Owing to prolonged immunodeficiency, transplant recipients are also at high risk of viral illness, particularly from reactivation of chronic viruses, such as cytomegalovirus (CMV). Although traditional antiviral drug treatment has clearly decreased the incidence of CMV disease, refractory or persistent CMV infections still occur in a subgroup of patients. Currently, adoptive immunotherapies with CMV-specific T cells have been developed for treating CMV infection. The clinical efficacy and possible mechanism have also been studied by Huang’s group.

A prospective study conducted at Peking University has enrolled 32 patients with refractory CMV infection who underwent adoptive CMV-specific T-cell infusion after haplo-HSCT^80^. In the refractory cohort, 27 of the 32 treated patients showed CMV clearance within 4 weeks after adoptive T-cell transfer without recurrence. The *in vivo* expansion of CMV-specific T cells and improvements in the cytokine production and proliferation ability of CMV-specific T cells were observed after adoptive therapy. In addition, decreased expression of programmed death-1 (PD-1) on CMV-specific T cells was observed. Nevertheless, in the remaining 5 patients who showed CMV recurrence after transfer, neither the quantity nor the function of CMV-specific T cells was restored. This study has provided important insights into the reconstitution of long-term antiviral immunity associated with the *in vivo* expansion and functional recovery of CMV-specific T cells. Subsequently, the efficacy of donor-derived CMV-specific cytotoxic T cells (CTLs) was explored as a first-line therapy for CMV infection after allo-HSCT^81^. In a humanized CMV-infected mouse model, CMV-specific CTLs have been found to effectively fight systemic CMV infection by promoting the restoration of graft-derived endogenous CMV-specific immunity *in vivo*. In the clinical cohort, first-line therapy with CTL significantly decreased the rates of persistent and late CMV infection. These outcomes suggest that adoptively infused CMV-CTL may stimulate the recovery of endogenous CMV-specific immunity.

As shown in this section, allo-HSCT is the curative choice for most hematologic malignancies through the GVL or GVT effect. Numerous studies have indicated that treating patients with hematological malignancies with haplo-HSCT can achieve comparable outcomes to those in patients who undergo ISD-HSCT. The advent of haplo-HSCT has led to a new era in which everyone has a donor, and the end of donor shortage is a first step potentially allowing HSCT to cure everyone. Currently, increasing evidence suggests that haploidentical allografts have a stronger GVL effect. Donor selection remains the future focus when haploidentical and ISDs are both available.

## Future perspectives

Each cellular immunotherapeutic strategy has achieved varying degrees of success in treating hematologic malignancies (**Table 5**). CAR-T cell therapy shows a significant, favorable, short-term response in B cell malignancy, but how to improve the long-term efficacy and translate this approach to other malignancies remains to be addressed. Future studies identifying exactly which genes enhance the persistence and expansion of T cells and exploring attractive targets in non-B cell malignancies are required for further optimization. NK cell-based immunotherapy has been attempted in a broad range of hematological malignancies, thus providing advantages of killing targets in a non-specific manner and being readily available for immediate use. However, the clinical efficacy is relatively limited. Advances in genetic engineering and *ex vivo* expansion techniques might help enhance the clinical efficacy.

**Table 5:** Current modalities of cellular immunotherapy: pros and cons

	Clinical indications		Disadvantages needed to be improved	
CAR-T-cell therapy	1.	This therapy elicits rapid and high response in refractory cases	1.	This therapy has non-durable efficacy
	2.	CAR-T therapy may be an effective way to induce remission and serve as a “bridge to transplant”	2.	Suitable targets as effective as CD19 in non-B-cell malignancies are lacking
			3.	Toxicity is unavoidable and unpredictable
NK cell-based therapy	1.	NK cells provide an “off-the-shelf” product and could be readily available for immediate clinical use	1.	The efficacy of adoptively transferred allogeneic NK cell infusion is relatively limited
	2.	NK cell-based therapy could be applied in refractory, MRD positive, or remission cases in non- transplant settings, and could complement HSCT	2.	CAR-modified NK cell therapy is largely in the preclinical phase
Allo-HSCT	This therapy is currently the only curative option for hematologic malignancy		1.	There is a certain incidence of transplant related mortality
			2.	GvHD might affect quality of life

Great advances in the field of stem cell transplantation, particularly from haploidentical allografts, have brought increasing therapeutic opportunities to patients with hematologic malignancy. In the context of HSCT, what will be the best role of the above novel cellular immunotherapies? Our best hypothesis is that these novel cellular immunotherapies will be applied as a complement to transplantation. For example, these novel strategies may be best suited to serve as a bridge to HSCT, to treat post-HSCT relapse, and as an option for transplant-ineligible patients. Consequently, the prognosis of hematologic cancers may be improved to the maximum extent through these advanced diversified approaches.
